# (*meso*-5,7,7,12,14,14-Hexamethyl-1,4,8,11-tetra­aza­cyclo­tetra­deca-4,11-diene)nickel(II) dibromide dihydrate

**DOI:** 10.1107/S1600536810033714

**Published:** 2010-09-30

**Authors:** XiuLi He, Feifei Shi, Yonghong Lu

**Affiliations:** a Ordered Matter Science Research Center, College of Chemistry and Chemical Engineering, Southeast University, Nanjing 210096, People’s Republic of China

## Abstract

The asymmetric unit of the title compound, [Ni(C_16_H_32_N_4_)]Br_2_·2H_2_O, consists of one half [Ni(C_16_H_32_N_4_)]^2+^ cation, one Br^−^ anion and one water mol­ecule of crystallization. The Ni^II^ ion lies on an inversion centre in a square-planar environment formed by the four macrocyclic ligand N atoms. In the crystal structure, the cations, anions and water mol­ecules are linked *via* inter­molecular N—H⋯Br and O—H⋯Br hydrogen bonds, forming discrete chains with set-graph motif *D*(2)*D*_2_^2^(7)*D*_2_^1^(3)*D*_3_^2^(8). The water mol­ecules and Br^−^ ions are linked with set-graph motif *R*_4_^2^(8).

## Related literature

For related structures, see: Ballester *et al.* (2000 ▶); Heinlein & Tebbe (1985 ▶); Shen *et al.* (1999 ▶); Szalda *et al.* (1989 ▶); Wang *et al.* (2007 ▶); Whimp *et al.* (1970 ▶); Yang (2005 ▶). For the preparation of the precursor complex C_16_H_32_N_4_·2HBr·2H_2_O, see: Hay *et al.* (1975 ▶). For hydrogen-bond motifs, see: Bernstein *et al.* (1995 ▶).
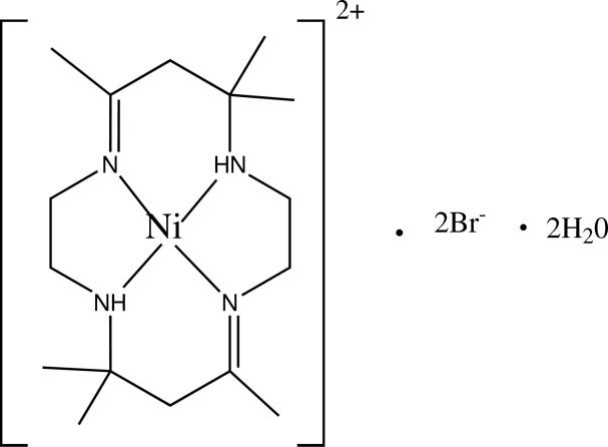


## Experimental

### 

#### Crystal data

[Ni(C_16_H_32_N_4_)]Br_2_·2H_2_O
*M* *_r_* = 535.02
Monoclinic, 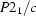

*a* = 8.0349 (16) Å
*b* = 15.619 (3) Å
*c* = 8.9355 (18) Å
β = 99.72 (3)°
*V* = 1105.3 (4) Å^3^
*Z* = 2
Mo *K*α radiation
μ = 4.51 mm^−1^
*T* = 293 K
0.27 × 0.20 × 0.20 mm

#### Data collection

Rigaku SCXmini diffractometer
Absorption correction: multi-scan (*CrystalClear*; Rigaku, 2005 ▶) *T*_min_ = 0.831, *T*_max_ = 0.862
11266 measured reflections
2531 independent reflections
1995 reflections with *I* > 2σ(*I*)
*R*_int_ = 0.048

#### Refinement

*R*[*F* ^2^ > 2σ(*F* ^2^)] = 0.044
*wR*(*F* ^2^) = 0.099
*S* = 1.05
2531 reflections
116 parameters
H-atom parameters constrained
Δρ_max_ = 0.74 e Å^−3^
Δρ_min_ = −0.74 e Å^−3^

### 

Data collection: *CrystalClear* (Rigaku, 2005 ▶); cell refinement: *CrystalClear*; data reduction: *CrystalClear*; program(s) used to solve structure: *SHELXS97* (Sheldrick, 2008 ▶); program(s) used to refine structure: *SHELXL97* (Sheldrick, 2008 ▶); molecular graphics: *SHELXTL* (Sheldrick, 2008 ▶); software used to prepare material for publication: *SHELXTL*.

## Supplementary Material

Crystal structure: contains datablocks I, global. DOI: 10.1107/S1600536810033714/bx2296sup1.cif

Structure factors: contains datablocks I. DOI: 10.1107/S1600536810033714/bx2296Isup2.hkl

Additional supplementary materials:  crystallographic information; 3D view; checkCIF report

## Figures and Tables

**Table 1:** Hydrogen-bond geometry (Å, °)

*D*—H⋯*A*	*D*—H	H⋯*A*	*D*⋯*A*	*D*—H⋯*A*
N1—H1*A*⋯Br1^i^	0.91	2.53	3.413 (3)	164
O1—H1*E*⋯Br1^ii^	0.85	2.53	3.374 (3)	169
O1—H1*F*⋯Br1^iii^	0.85	2.55	3.388 (5)	170

Symmetry codes: (i) 
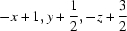
; (ii) 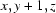; (iii) 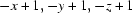.

**Fig. 1.**
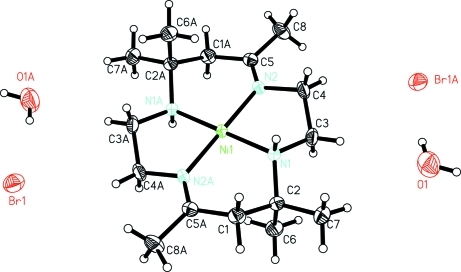
The asymmetric unit of (I), showing the atom-numbering scheme. Displacement ellipsoids are drawn at the 30% probability level. Unlabeled atoms are related to labeled atoms by the symmetry code (1-x,1-y,1-z)

**Fig. 2.**
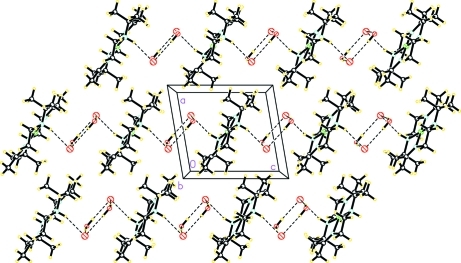
View of (I) along b, showing chains of organic cations running parallel to the (001) direction, with Br- anions and water molecules lying between them.

## supplementary crystallographic information

### Comment

The structures of several related macrocyclic complexes have been reported (Whimp *et al.*,1970; Yang, 2005; Heinlein *et al.*, 1985) for many times in the past years. The nickel(II) tetraazamacrocyclic complex cation, [Ni(C_16_H_32_N_4_)]^2+^ has both *meso* and enantiomeric forms and can combine with different anions to form many kinds of structures (Shen *et al.*, 1999; Ballester *et al.*, 2000; Wang *et al.*, 2007). We herein report the crystal structure of a new compound synthesized by reaction of Ni(CH_3_COO)_2_.4H_2_O and the complex C_16_H_32_N_4_.2HBr.2H_2_O in methanol solution. As shown in Fig.1, the Ni^II ^atom is coordinated by four N atoms from the tetraazamacrocycle in a square-planar geometry. The metal atom and the four N atoms are coplanar. The Ni—*N*(amine) and *N*(imine) bond distances are 1.934 (3) Å and 1.916 (3)Å and are similar to those in previously report (Szalda *et al.*,1989). In the crystal structure, the cations, anions and water molecules are linked via intermolecular N—H···Br and O— H···Br hydrogen bonds forming discrete chains with set-graph motif D_1_^1^(2), D_2_^2^(7), D_2_^1^(3) and D_3_^2^(8). The water molecules and Br^–^ ions are linked forming R_4_^2^(8) set-graph motif (Bernstein *et al.*, 1995), Fig 2.

### Experimental

All chemicals were of reagent grade and were used as received without further purification. The precursor complex C_16_H_32_N_4 _.2HBr.2H_2_O was prepared by described in the literature method (Hay *et al.*, 1975). To a 10 ml methanol solution of Ni(CH_3_COO)_2_.4H_2_O (0.2 mmol, 0.049 g), a 5 ml methanol solution of C_16_H_32_N_4_.2HBr.2H_2_O (0.2 mmol, 0.0957 g) was added dropwise with stirring. The resulting solution was continuously stirred for about 30 min. Yellow crystals suitable for X-ray analysis were obtained by slow evaporation at room temperature over several days.

### Refinement

All hydrogen atoms were placed in calculated positions with C—H = 0.96 to 0.97 Å, N—H = 0.91 Å and O—H = 0.82 Å. They were included in the refinement in the riding-motion approximation with U_iso_(H) = 1.2U_eq_(C, N, O) or U_iso_(H) = 1.5U_eq_(C_methyl_).

### Crystal data

**Table:**

[Ni(C_16_H_32_N_4_)]Br_2_·2H_2_O	*F*(000) = 548
*M**_r_* = 535.02	*D*_x_ = 1.608 Mg m^−^^3^
Monoclinic, *P*2_1_/*c*	Mo *K*α radiation, λ = 0.71073 Å
Hall symbol: -P 2ybc	Cell parameters from 3476 reflections
*a* = 8.0349 (16) Å	θ = 2.3–27.5°
*b* = 15.619 (3) Å	µ = 4.51 mm^−^^1^
*c* = 8.9355 (18) Å	*T* = 293 K
β = 99.72 (3)°	Prism, brown
*V* = 1105.3 (4) Å^3^	0.27 × 0.20 × 0.20 mm
*Z* = 2	

### Data collection

**Table:**

Rigaku SCXmini diffractometer	2531 independent reflections
Radiation source: fine-focus sealed tube	1995 reflections with *I* > 2σ(*I*)
graphite	*R*_int_ = 0.048
Detector resolution: 13.6612 pixels mm^-1^	θ_max_ = 27.5°, θ_min_ = 3.4°
thin–slice ω scans	*h* = −10→10
Absorption correction: multi-scan (*CrystalClear*; Rigaku, 2005)	*k* = −20→20
*T*_min_ = 0.831, *T*_max_ = 0.862	*l* = −11→11
11266 measured reflections	

### Refinement

**Table:**

Refinement on *F*^2^	Primary atom site location: structure-invariant direct methods
Least-squares matrix: full	Secondary atom site location: difference Fourier map
*R*[*F*^2^ > 2σ(*F*^2^)] = 0.044	Hydrogen site location: inferred from neighbouring sites
*wR*(*F*^2^) = 0.099	H-atom parameters constrained
*S* = 1.05	*w* = 1/[σ^2^(*F*_o_^2^) + (0.0317*P*)^2^ + 1.6713*P*] where *P* = (*F*_o_^2^ + 2*F*_c_^2^)/3
2531 reflections	(Δ/σ)_max_ < 0.001
116 parameters	Δρ_max_ = 0.74 e Å^−^^3^
0 restraints	Δρ_min_ = −0.74 e Å^−^^3^

### Special details

**Table:**

Geometry. All e.s.d.'s (except the e.s.d. in the dihedral angle between two l.s. planes) are estimated using the full covariance matrix. The cell e.s.d.'s are taken into account individually in the estimation of e.s.d.'s in distances, angles and torsion angles; correlations between e.s.d.'s in cell parameters are only used when they are defined by crystal symmetry. An approximate (isotropic) treatment of cell e.s.d.'s is used for estimating e.s.d.'s involving l.s. planes.
Refinement. Refinement of *F*^2^ against ALL reflections. The weighted *R*-factor *wR* and goodness of fit *S* are based on *F*^2^, conventional *R*-factors *R* are based on *F*, with *F* set to zero for negative *F*^2^. The threshold expression of *F*^2^ > σ(*F*^2^) is used only for calculating *R*-factors(gt) *etc*. and is not relevant to the choice of reflections for refinement. *R*-factors based on *F*^2^ are statistically about twice as large as those based on *F*, and *R*- factors based on ALL data will be even larger.

### Fractional atomic coordinates and isotropic or equivalent isotropic displacement parameters (Å^2^)

**Table:**

	*x*	*y*	*z*	*U*_iso_*/*U*_eq_	
Ni1	0.5000	0.5000	0.5000	0.02894 (16)	
Br1	0.69586 (6)	0.10330 (3)	0.63849 (6)	0.06334 (18)	
C1	0.8076 (5)	0.5280 (2)	0.7876 (4)	0.0410 (8)	
H1B	0.9228	0.5334	0.8406	0.061*	
H1C	0.7344	0.5312	0.8633	0.061*	
C2	0.7698 (4)	0.6042 (2)	0.6830 (4)	0.0362 (8)	
C3	0.5246 (5)	0.6761 (2)	0.5232 (5)	0.0546 (11)	
H3A	0.5877	0.6831	0.4405	0.065*	
H3B	0.5384	0.7273	0.5854	0.065*	
C4	0.3413 (5)	0.6610 (2)	0.4621 (5)	0.0536 (11)	
H4A	0.2748	0.6656	0.5429	0.064*	
H4B	0.3003	0.7030	0.3846	0.064*	
C5	0.2117 (4)	0.5591 (2)	0.2827 (4)	0.0343 (7)	
C6	0.8765 (5)	0.6034 (3)	0.5571 (5)	0.0503 (10)	
H6A	0.8525	0.5525	0.4972	0.075*	
H6B	0.9941	0.6044	0.6013	0.075*	
H6C	0.8503	0.6528	0.4937	0.075*	
C7	0.8061 (5)	0.6849 (3)	0.7812 (5)	0.0561 (11)	
H7A	0.7833	0.7348	0.7184	0.084*	
H7B	0.9225	0.6851	0.8290	0.084*	
H7C	0.7353	0.6853	0.8576	0.084*	
C8	0.0896 (5)	0.6244 (3)	0.2077 (5)	0.0528 (10)	
H8A	0.0147	0.5984	0.1252	0.079*	
H8B	0.1504	0.6702	0.1695	0.079*	
H8C	0.0252	0.6468	0.2802	0.079*	
N1	0.5857 (3)	0.60021 (16)	0.6151 (3)	0.0332 (6)	
H1A	0.5300	0.6015	0.6957	0.040*	
N2	0.3276 (3)	0.57404 (17)	0.3970 (3)	0.0333 (6)	
O1	0.6240 (6)	0.8913 (2)	0.6616 (5)	0.0923 (12)	
H1E	0.6525	0.9438	0.6665	0.111*	
H1F	0.5421	0.8854	0.5885	0.111*	

### Atomic displacement parameters (Å^2^)

**Table:**

	*U*^11^	*U*^22^	*U*^33^	*U*^12^	*U*^13^	*U*^23^
Ni1	0.0263 (3)	0.0225 (3)	0.0368 (3)	0.0004 (2)	0.0016 (2)	0.0001 (3)
Br1	0.0521 (3)	0.0655 (3)	0.0752 (4)	−0.0076 (2)	0.0189 (2)	−0.0112 (2)
C1	0.041 (2)	0.046 (2)	0.0338 (19)	0.0010 (16)	−0.0019 (16)	−0.0021 (16)
C2	0.0299 (17)	0.0349 (19)	0.042 (2)	−0.0014 (14)	0.0011 (15)	−0.0035 (15)
C3	0.057 (3)	0.0238 (17)	0.075 (3)	−0.0031 (17)	−0.012 (2)	0.0055 (19)
C4	0.050 (2)	0.0277 (19)	0.074 (3)	0.0112 (17)	−0.013 (2)	−0.0055 (19)
C5	0.0321 (17)	0.0398 (19)	0.0319 (18)	0.0022 (14)	0.0079 (14)	0.0059 (15)
C6	0.043 (2)	0.051 (2)	0.061 (3)	−0.0022 (18)	0.021 (2)	0.008 (2)
C7	0.051 (2)	0.041 (2)	0.071 (3)	−0.0062 (18)	−0.004 (2)	−0.012 (2)
C8	0.048 (2)	0.057 (3)	0.048 (2)	0.019 (2)	−0.0043 (19)	0.003 (2)
N1	0.0299 (14)	0.0264 (14)	0.0430 (17)	−0.0001 (11)	0.0051 (12)	−0.0013 (12)
N2	0.0321 (15)	0.0257 (14)	0.0407 (16)	0.0040 (11)	0.0025 (13)	0.0021 (12)
O1	0.103 (3)	0.070 (2)	0.097 (3)	0.013 (2)	−0.001 (2)	0.019 (2)

### Geometric parameters (Å, °)

**Table:**

Ni1—N2	1.916 (3)	C4—H4B	0.9700
Ni1—N2^i^	1.916 (3)	C5—N2	1.282 (4)
Ni1—N1	1.934 (3)	C5—C8	1.494 (5)
Ni1—N1^i^	1.934 (3)	C5—C1^i^	1.494 (5)
C1—C5^i^	1.494 (5)	C6—H6A	0.9600
C1—C2	1.513 (5)	C6—H6B	0.9600
C1—H1B	0.9700	C6—H6C	0.9600
C1—H1C	0.9700	C7—H7A	0.9600
C2—N1	1.503 (4)	C7—H7B	0.9600
C2—C6	1.525 (5)	C7—H7C	0.9600
C2—C7	1.535 (5)	C8—H8A	0.9600
C3—N1	1.477 (4)	C8—H8B	0.9600
C3—C4	1.501 (5)	C8—H8C	0.9600
C3—H3A	0.9700	N1—H1A	0.9100
C3—H3B	0.9700	O1—H1E	0.8500
C4—N2	1.475 (5)	O1—H1F	0.8500
C4—H4A	0.9700		
			
N2—Ni1—N2^i^	180.000 (1)	N2—C5—C1^i^	120.7 (3)
N2—Ni1—N1	86.01 (12)	C8—C5—C1^i^	114.8 (3)
N2^i^—Ni1—N1	93.99 (12)	C2—C6—H6A	109.5
N2—Ni1—N1^i^	93.99 (12)	C2—C6—H6B	109.5
N2^i^—Ni1—N1^i^	86.01 (12)	H6A—C6—H6B	109.5
N1—Ni1—N1^i^	180.0	C2—C6—H6C	109.5
C5^i^—C1—C2	117.5 (3)	H6A—C6—H6C	109.5
C5^i^—C1—H1B	107.9	H6B—C6—H6C	109.5
C2—C1—H1B	107.9	C2—C7—H7A	109.5
C5^i^—C1—H1C	107.9	C2—C7—H7B	109.5
C2—C1—H1C	107.9	H7A—C7—H7B	109.5
H1B—C1—H1C	107.2	C2—C7—H7C	109.5
N1—C2—C1	107.3 (3)	H7A—C7—H7C	109.5
N1—C2—C6	109.9 (3)	H7B—C7—H7C	109.5
C1—C2—C6	111.7 (3)	C5—C8—H8A	109.5
N1—C2—C7	110.1 (3)	C5—C8—H8B	109.5
C1—C2—C7	107.1 (3)	H8A—C8—H8B	109.5
C6—C2—C7	110.6 (3)	C5—C8—H8C	109.5
N1—C3—C4	106.8 (3)	H8A—C8—H8C	109.5
N1—C3—H3A	110.4	H8B—C8—H8C	109.5
C4—C3—H3A	110.4	C3—N1—C2	113.8 (3)
N1—C3—H3B	110.4	C3—N1—Ni1	107.4 (2)
C4—C3—H3B	110.4	C2—N1—Ni1	119.1 (2)
H3A—C3—H3B	108.6	C3—N1—H1A	105.1
N2—C4—C3	106.9 (3)	C2—N1—H1A	105.1
N2—C4—H4A	110.3	Ni1—N1—H1A	105.1
C3—C4—H4A	110.3	C5—N2—C4	118.5 (3)
N2—C4—H4B	110.3	C5—N2—Ni1	129.9 (2)
C3—C4—H4B	110.3	C4—N2—Ni1	111.5 (2)
H4A—C4—H4B	108.6	H1E—O1—H1F	108.1
N2—C5—C8	124.5 (3)		
			
C5^i^—C1—C2—N1	64.1 (4)	N2—Ni1—N1—C2	−154.2 (3)
C5^i^—C1—C2—C6	−56.4 (4)	N2^i^—Ni1—N1—C2	25.8 (3)
C5^i^—C1—C2—C7	−177.6 (3)	N1^i^—Ni1—N1—C2	79 (100)
N1—C3—C4—N2	−48.4 (5)	C8—C5—N2—C4	1.2 (5)
C4—C3—N1—C2	179.0 (3)	C1^i^—C5—N2—C4	180.0 (3)
C4—C3—N1—Ni1	45.0 (4)	C8—C5—N2—Ni1	−176.6 (3)
C1—C2—N1—C3	173.5 (3)	C1^i^—C5—N2—Ni1	2.2 (5)
C6—C2—N1—C3	−64.9 (4)	C3—C4—N2—C5	−148.4 (4)
C7—C2—N1—C3	57.2 (4)	C3—C4—N2—Ni1	29.8 (4)
C1—C2—N1—Ni1	−58.4 (3)	N2^i^—Ni1—N2—C5	−65 (100)
C6—C2—N1—Ni1	63.2 (3)	N1—Ni1—N2—C5	173.9 (3)
C7—C2—N1—Ni1	−174.7 (3)	N1^i^—Ni1—N2—C5	−6.1 (3)
N2—Ni1—N1—C3	−23.2 (3)	N2^i^—Ni1—N2—C4	117 (100)
N2^i^—Ni1—N1—C3	156.8 (3)	N1—Ni1—N2—C4	−4.0 (3)
N1^i^—Ni1—N1—C3	−150 (100)	N1^i^—Ni1—N2—C4	176.0 (3)

Symmetry codes: (i) −*x*+1, −*y*+1, −*z*+1.

### Hydrogen-bond geometry (Å, °)

**Table:**

*D*—H···*A*	*D*—H	H···*A*	*D*···*A*	*D*—H···*A*
N1—H1A···Br1^ii^	0.91	2.53	3.413 (3)	164
O1—H1E···Br1^iii^	0.85	2.53	3.374 (3)	169
O1—H1F···Br1^i^	0.85	2.55	3.388 (5)	170

Symmetry codes: (ii) −*x*+1, *y*+1/2, −*z*+3/2; (iii) *x*, *y*+1, *z*; (i) −*x*+1, −*y*+1, −*z*+1.
